# Seasonally lagged effects of climatic factors on malaria incidence in South Africa

**DOI:** 10.1038/s41598-017-02680-6

**Published:** 2017-05-29

**Authors:** Takayoshi Ikeda, Swadhin K. Behera, Yushi Morioka, Noboru Minakawa, Masahiro Hashizume, Ataru Tsuzuki, Rajendra Maharaj, Philip Kruger

**Affiliations:** 1 Japan Agency for Marine-Earth Science and Technology, Yokohama Institute for Earth Sciences, 3173-25 Showa-machi, Kanazawa-ku, Yokohama 236-0001 Japan; 2 Nagasaki University, Institute of Tropical Medicine, 1-12-4 Sakamoto, Nagasaki, 852-8523 Japan; 3 Malaria Research Programme, Medical Research Council, Ridge Road, Durban, 4067 South Africa; 4 Malaria Control Programme, Limpopo Department of Health, Voortrekker Street, Tzaneen, Limpopo 0850 South Africa

## Abstract

Globally, malaria cases have drastically dropped in recent years. However, a high incidence of malaria remains in some sub-Saharan African countries. South Africa is mostly malaria-free, but northeastern provinces continue to experience seasonal outbreaks. Here we investigate the association between malaria incidence and spatio-temporal climate variations in Limpopo. First, dominant spatial patterns in malaria incidence anomalies were identified using self-organizing maps. Composite analysis found significant associations among incidence anomalies and climate patterns. A high incidence of malaria during the pre-peak season (Sep-Nov) was associated with the climate phenomenon La Niña and cool air temperatures over southern Africa. There was also high precipitation over neighbouring countries two to six months prior to malaria incidence. During the peak season (Dec-Feb), high incidence was associated with positive phase of Indian Ocean Subtropical Dipole. Warm temperatures and high precipitation in neighbouring countries were also observed two months prior to increased malaria incidence. This lagged association between regional climate and malaria incidence suggests that in areas at high risk for malaria, such as Limpopo, management plans should consider not only local climate patterns but those of neighbouring countries as well. These findings highlight the need to strengthen cross-border control of malaria to minimize its spread.

## Introduction

Malaria is the deadliest vector-borne disease affecting people worldwide, with nearly half of the world’s population at risk. In 2015, malaria was responsible for more than 200 million cases and 438,000 deaths^1^. In recent years, global incidence has dropped drastically, however there is still high incidence of malaria in sub-Saharan African countries, summing to nearly 90% of all cases^1^. Thus, research to improve both the prediction of outbreaks and the management of malaria remains vital to decrease the burden of the disease in these countries. South Africa is one of the few countries in the sub-Saharan region that is mostly malaria-free, however, the northeastern provinces, such as Limpopo, are still experiencing a high incidence of malaria^2, 3^.

Several factors are associated with the seasonal and interannual variations of malaria incidences. Climate variation is a major factor. In Limpopo, climate variations range from tropical to subtropical. The landscape in Limpopo is diverse with farmlands, forests, mountains, grasslands, and riparian areas. The province is in a high-risk malaria transmission zone because it borders Mozambique, Zimbabwe and Botswana, countries that still have high malaria incidence throughout the year^1^.

The annual malaria incidence link to sea surface temperature (SST) associated with climate variation has been described previously^4^ and malaria incidence has been linked to remote influences such as the monsoon rainfalls^5^ and large-scale climate phenomena, such as the Indian Ocean Dipole^6–8^ and El Niño-Southern Oscillation^9, 10^. These studies suggest that remote large-scale climate phenomena could influence local malaria incidence via atmospheric teleconnection.

Such teleconnections could theoretically pass a signal from the source region in the tropical Pacific via mid-latitude wave guides and affect regions of sub-Saharan Africa by modifying local weather and climatic conditions^11^. For example, the recent El Niño of 2015–2016 had a terrible impact on agriculture production in South Africa as well as Ethiopia^12^. The other important mode of climate variability in the southern Indian Ocean is the so-called Indian Ocean Subtropical Dipole (IOSD), which directly affects southwestern Africa by modifying the moisture transported to that region by altering basin-scale atmospheric circulations^13, 14^. However, the potential relationship between these large-scale phenomena and malarial incidence have not been investigated on a seasonal and interannual time scales for Limpopo. Additional teleconnections that could affect sub-Saharan Africa are El Niño and La Niña. Previous studies have reported relationships between malaria and climate variables including rainfall, humidity and temperature for African^15–22^ and Asian countries^23–25^.

Previous studies have shown that some climate factors are more important than others. For example, Weiss *et al*.^26^ used an air temperature suitability index to show that temperature across the entire African continent was a strong predictor for transmission of the malaria parasite *Plasmodium falciparum*. Other studies have reported that rainfall is a main driver of malaria incidence^22, 27^. Komen *et al*.^28^ showed that incidence rates in Limpopo were related to both temperature and rainfall. However, they also showed that temperature plays a more important role in influencing malaria transmission compared to rainfall^28^. Precipitation and temperature are the most commonly tested climate variables. However, Jury and Kanemba^29^ reported that zonal winds over the Western Pacific and Australia could also be associated with increased malaria incidences over South Africa. In addition to relationships with climate factors, a reasonable lag time between climate variables and malarial incidence is two months, with climate preceding malaria incidence. This is reasonable when considering the time required for the life cycles of the mosquito^30^ and parasite^31^, and the number of days between date of malarial diagnosis and date of entry into the system^32^. However, there have been reports of lags greater than two months^6, 19, 22^.

In this study, we used spatially explicit malaria case data to analyse the relationship between both local climatic effects and remote atmospheric teleconnections on the incidence of malaria in Limpopo, including potential lag effects.

## Results

**Figure 1:**
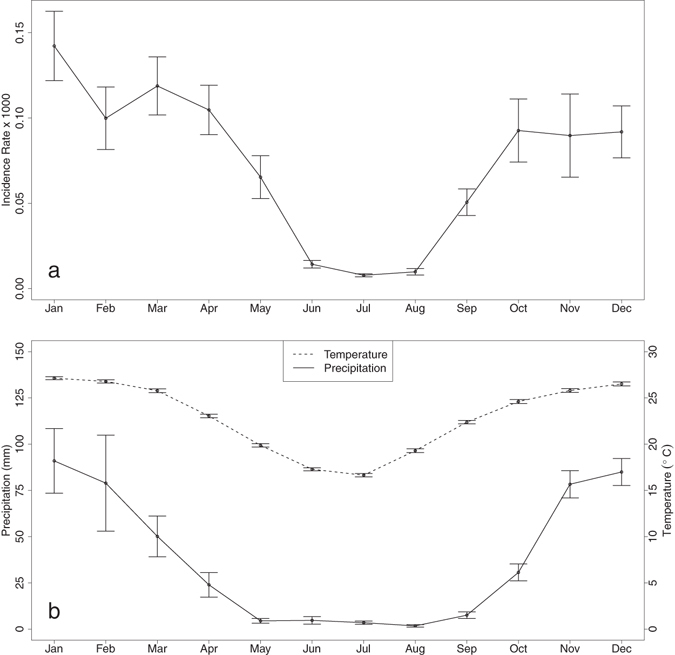
Monthly mean and standard deviation of (**a**) malaria incidence rate (% per 1000 person-years), (**b**) precipitation (solid line, mm month^−1^), and mean temperature (dashed line, °C) in Limpopo for 1998 to 2014. Data were obtained from the spatially-averaged grid of Limpopo from CRU TS 3.23. Figure was made in R version 3.2.2 (https://cran.r-project.org/).

**Figure 2:**
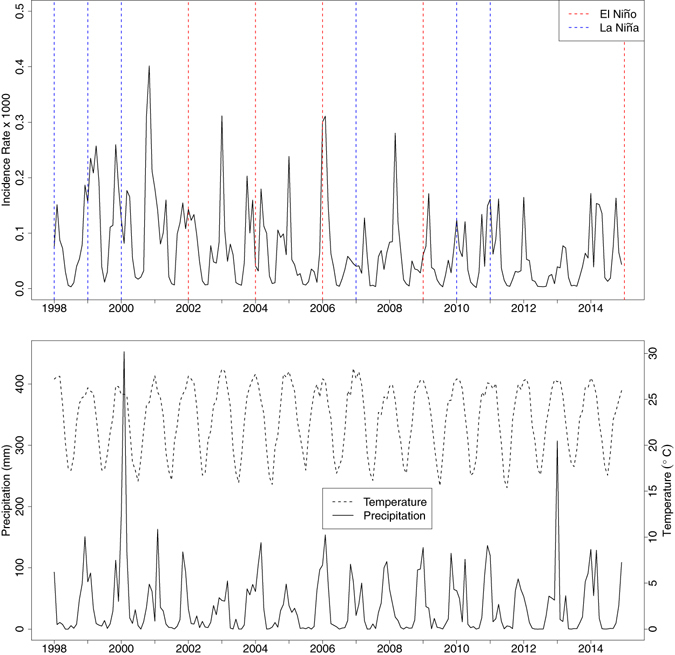
Time series of (**a**) malaria incidence rate (% per 1000 person-years), (**b**) precipitation (solid line, mm month^−1^), and mean temperature (dashed line, °C) by month for Limpopo from 1998 to 2014. Years are labeled with El Niño (red lines) and La Niña (blue lines) events. Figure was made in R version 3.2.2 (https://cran.r-project.org/).

Malaria incidence peaks in January over Limpopo (Fig. 1). The lowest malaria burden is usually observed during the dry and cold season (June to August; Figs 1 and 2) while the highest burden is observed during the warm and wet season (December to February). The years with the largest malaria burden are 2001, 2003, 2006 and 2008 (Fig. 2).

**Figure 3:**
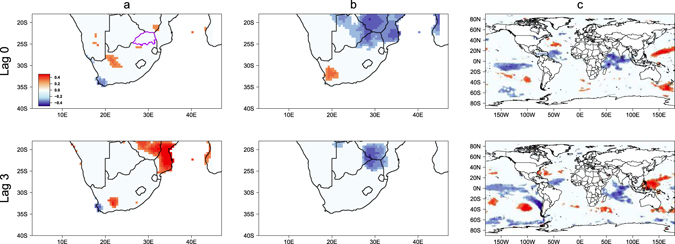
Correlation maps of seasonal malaria incidence in Limpopo and (**a**) precipitation, (**b**) mean temperature, and (**c**) SST anomalies. The top (bottom) row is associated to lags of zero (three) months. The climatic variables are leading the malaria incidence. Correlation coefficients at the 95% confidence level are shown. Figure was made in R version 3.2.2 (https://cran.r-project.org/).

To test whether malaria incidence rate anomalies were associated with local and regional climate factors, and large-scale climate phenomena, we first investigated the relationship among monthly malaria incidence of Limpopo and temperature, precipitation, and SST anomalies. Lagged correlation analyses (Fig. 3) are used to understand the gradual progression of those relationships and by doing so we recognize the possible mechanisms through which climate influences malaria incidence in Limpopo. Positive correlation is shown between precipitation and malaria incidence rate (MIR) anomalies over southern Mozambique at three-month lag (Fig. 3a). In contrast, Fig. 3b shows that there is a negative correlation with mean temperature anomalies over Limpopo and north of Limpopo at zero- and three-month lags. Figure 3c shows that MIR and SST anomalies have significant negative correlations in the tropical eastern Pacific off the coasts of Peru, indicating that high incidence rates tend to be associated with La Niña. Hence, we can deduce that significant statistical associations exist between seasonal MIR anomalies in Limpopo and regional (such as rainfall over neighbouring Mozambique) and remote climate factors (such as El Niño and La Niña).

**Figure 4:**
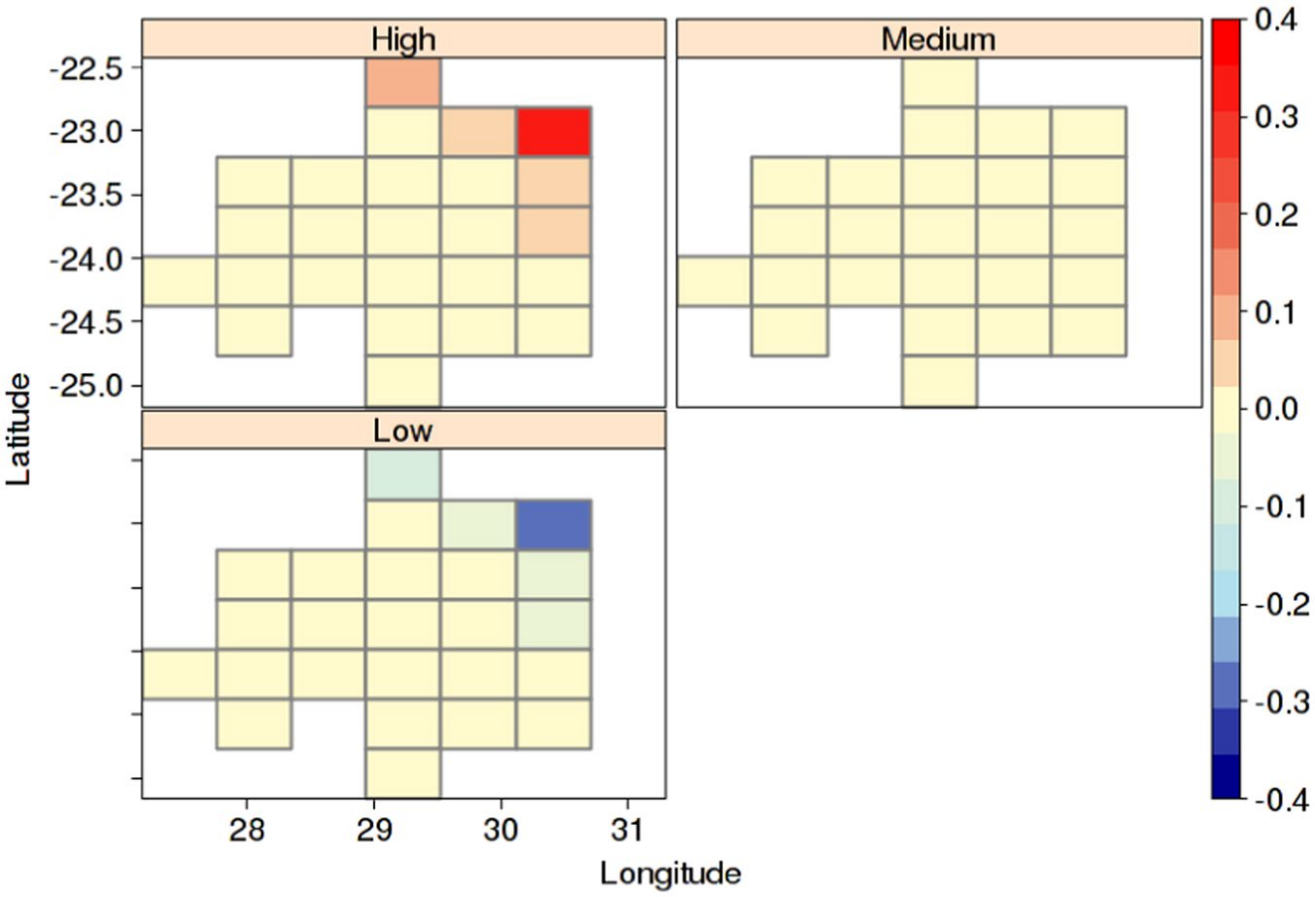
Self-organized map for malaria incidence rate anomalies (% per 1000 person-years) in Limpopo. Cells represent local municipalities of Limpopo, with coloured cells being Musina, Thulamela, Mutale, Greater Giyani, and Ba-Phalaborwa. Figure was made in R version 3.2.2 (https://cran.r-project.org/).

**Figure 5:**
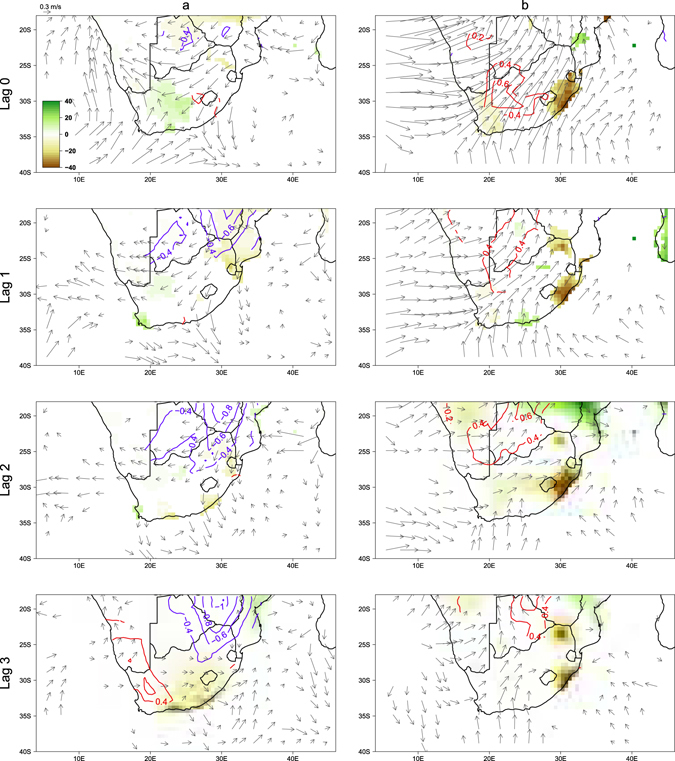
Composites of precipitation (shade, mm month^−1^), mean temperature (contour, °C), and wind (vector, m s^−1^) in southern Africa for (**a**) SON and (**b**) DJF. Differences in composite anomalies between the high and low malaria incidence years are shown. Rows associate to lags of zero to three months. Composites at 90% confidence level are shown. Figure was made in R version 3.2.2 (https://cran.r-project.org/).

**Figure 6:**
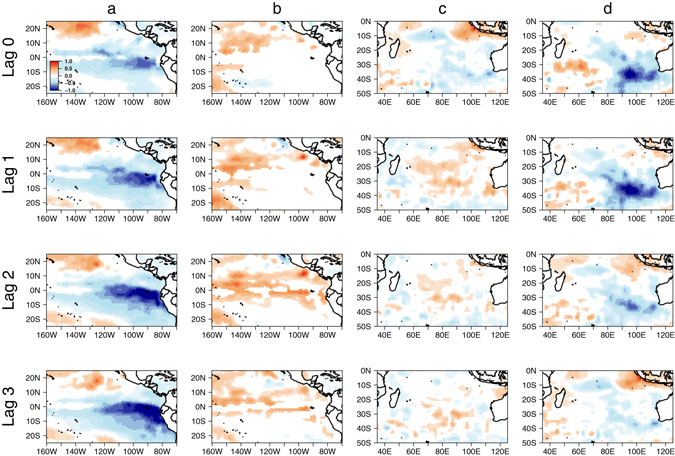
Composites of SST (°C) in the tropical eastern Pacific for (**a**) SON and (**b**) DJF. Likewise for the Indian Ocean for (**c**) SON and (**d**) DJF. Differences in composite anomalies between the high and low malaria incidence years are shown. Rows associate to lags of zero to three months. Composites at 90% confidence level are shown. Figure was made in R version 3.2.2 (https://cran.r-project.org/).

To further investigate the mechanism that links malaria incidence with climate factors, SOM analysis was performed to first identify important patterns in malaria incidence anomaly. Three types of incidence patterns, labelled as high, medium and low events were found (Fig. 4). Maximum variability was seen in five cells, which corresponds to the municipalities Musina, Thulamela, Mutale, Greater Giyani and Ba-Phalaborwa in the Vhembe and Mopani districts. These municipalities border Mozambique, Zimbabwe and South Africa’s Kruger National Park, and are known to have large historical incidence rates of malaria^32^. For each season, we created composite maps for precipitation, mean temperature, and wind anomalies by taking the difference in composite anomalies between the high and low malaria incidence years (Fig. 5). Composite maps for the pre-peak season (September to November) show that precipitation was not a significant factor throughout the season (Fig. 5a). However, wetter conditions were observed in September at two-month lag over southeastern Mozambique (Supplementary Fig. S1a) with visible La Niña patterns (Supplementary Fig. S1b). Six-months prior to the pre-peak season, higher than normal precipitation was seen over much of Limpopo, southern Zimbabwe and Mozambique with winds blowing westward coming from the Mozambique Channel and Indian Ocean (Supplementary Fig. S2). Mean temperature was lower than average in the pre-peak season at all lags (Fig. 5a). We also plotted composite maps for SST anomalies in the tropical eastern Pacific off coasts of Peru (Fig. 6a,b) and in the Indian Ocean (Fig. 6c,d) to show the related impacts of large-scale climate phenomena. These regional climate patterns coincided with significant La Niña patterns in the tropical eastern Pacific (Fig. 6a), which are known to be responsible for wetter than normal austral summers in South Africa^33^.

Composite maps for the peak of the malaria season (December to February) were also created for the same variables. Figure 5b shows that MIR anomalies were associated with higher than normal precipitation in southern Mozambique at a lag of two months. Unlike the SON season, southwesterly wind anomalies were found over Limpopo and most of southern Africa but only at lag 0. In addition, higher than normal mean temperature was observed at most lags. However, a striking difference is seen in SST anomalies, with no La Niña pattern visible (Fig. 6b). Instead, positive IOSD patterns (Fig. 6d), which were not seen in SON (Fig. 6c) were associated with above normal rainfall in southern Africa^13^ explaining MIR anomalies.

## Discussion

We have shown that there are seasonal lagged effects of climatic factors from local regions and neighbouring countries on the timing and severity of malaria outbreaks in Limpopo. Results from the correlation analysis have shown that there were significant associations between malaria incidence and climate variables, however this alone cannot explain the causality of the incidences. For this reason, we have also used composite analysis to further investigate whether there were associations between high malaria incidence and climate variables during pre-peak and peak malaria seasons. A high association was observed among La Niña, the IOSD and the incidence rates of malaria at several monthly lags. In the peak malaria season, higher local rainfall and rainfall in neighbouring countries such as Mozambique was three to four times higher than normal in dry winter months (sometimes these months experience as little as 10-mm of rain in a month^34^). This unusually wet dry season may have created favourable habitats and breeding conditions for mosquitoes such as *Anopheles arabiensis*, one of the primary malaria vectors in South Africa. This finding corroborates that precipitation acts as the main driver for malaria incidence^6, 15^. Mosquitoes are most abundant during the wet season, whereas in the dry season, numbers remain low because there is insufficiency to support the insect’s early aquatic life stages^35^. Although composite maps for the pre-peak malaria season did not show significant patterns of higher than normal rainfall, there were significant La Niña patterns for this season. La Niña conditions in July may explain the wetter conditions recorded along the southern coast of Mozambique in September (Supplementary Fig. S1). Although the season was not wetter than normal, higher than normal rain in September may have provided sufficient water for mosquito breeding. In addition, the higher than normal precipitation six months prior to high incidence rates of malaria (Supplementary Fig. S2) could explain the increased number of mosquitoes surviving the austral winter season. Although winter months drop to less than 18 °C, it has been reported that *An*. *arabiensis* can feed, lay eggs, and enter diapause during winter^36, 37^.

The study also suggests strong association between temperature and high incidence of malaria but only when precipitation is also higher than normal. When malarial incidence was high during the peak season, the wetter and warmer conditions may have provided a more ideal environment for the mosquitoes to breed. However, it is important to note that when ambient temperatures are above a certain threshold, higher than normal temperatures can have a negative impact on mosquito abundance^30, 38^. On the other hand, in pre-peak season, the cooler than normal temperatures may have contributed to higher transmission rates^38^.

Zonal wind has been reported as an important driver in increasing malaria cases in Limpopo in a previous study^29^. However, this study examined annual malaria incidence rate and zonal wind at six-month lag, while using a simple method to identify high and low year events. Our results build on this work by investigating the relationship between malaria incidence anomalies and climate variables at a seasonal time scale, focusing on spatial variations of climate factors, and the occurrence of large-scale climate phenomena such as La Niña and the IOSD. In general, periods with higher malaria incidence in the pre-peak and peak seasons were preceded by La Niña patterns and positive IOSD patterns, respectively. Our results support previous studies that linked malaria incidence to large-scale climate phenomena, but shows for the first time the potential importance of regional and basin-scale climate patterns when comparing between seasons.

Results also suggest the importance of climate variables in neighbouring countries when assessing malarial incidence; climate in neighbouring countries was positively associated with malaria incidence anomalies in northeastern Limpopo.

Malaria in South Africa is still an ongoing issue that requires physical, social, economic and political considerations^39^. In addition to the relationship between incidence and climate variables discussed in this paper, immigrants and tourists from southern Mozambique may carry the malarial parasite into South Africa. Hence, there is a need for strengthening cross-border malaria control management to minimize the spread of malaria. Future work should focus on the potential effects of the seasonal fluctuations of malaria cases in neighbouring endemic countries such as Mozambique and Zimbabwe.

Our analyses sometimes showed two peaks (SON and DJF) in high malaria incidences within the same year (in 2000, 2004, and 2010). However, due to the low number of cases, we could not analyse the composite patterns of those events due to the lack of statistical significance. As more data become available, SOM analyses will be able to classify more patterns of incidence rate anomalies, enabling composite signals to be verified with statistical tests at higher significance levels. Nevertheless, in this study, composite patterns did not vary greatly when the analysis was limited to Limpopo, thereby suggesting robustness in our analysis.

In conclusion, our findings indicate that the effects of climatic factors on malaria incidences in Limpopo vary by season and location. We also found significant associations between large-scale climate phenomena and malaria incidences in Limpopo. This information could be useful when designing and/or initializing dynamical malaria models^40, 41^. This could improve malaria epidemic model predictions, possibly several months in advance, and thus form a valuable basis toward development of an early warning system for malaria in South Africa.

## Material and Methods

### Malaria case data

Malaria case data for Limpopo, South Africa were collected from January 1998 to December 2014, in which notifications were recorded each day. Records also included demographic information about the patient, such as age, sex, diagnosis date, residential address (out of five districts and 25 local municipalities), type of parasite (*P*. *falciparum and P*. *vivax*) and the source (local or imported). For further details on the data, refer to Gerritsen *et al*.^32^. For this study, individual cases were aggregated by season (SON, DJF, MAM, and JJA) for each municipality and this was multiplied by 1000 to obtain incidence rate per 1000 person-years. Incidence rates of each municipality were calculated by dividing case totals by the municipality’s population at risk, which was assumed to be the entire population. Population data were obtained from Statistics South Africa for census years^42^, and linearly interpolated to obtain estimates for all years. Slight variations in the interpolated values (that can be caused by actual variations in the population density) do not affect the results.

### Methods of analysis

For correlation analysis, we investigated variation in sliding seasonal averages of incidence rate anomalies in comparison to seasonally averaged surface air temperature and precipitation over southern Africa, and global SST anomalies at zero- and three-month lags (with climate conditions leading malaria incidence). Anomalies were defined as the residual value after subtracting the linear trend and monthly mean per local municipality for years 1998 to 2014. The residual value would represent the remaining signal that cannot be explained by the seasonal and trend effects. Pearson’s product moment correlation coefficients were calculated for each grid point, and tested at the 5% significance level with a two-sided test for association. Only significant correlation coefficients are shown in correlation plots.

To make use of both the spatial and temporal information of malaria incidence, Self-Organizing Maps (SOMs) were used. A SOM, introduced by Kohonen^43, 44^, is an unsupervised artificial neural network that extracts information from patterns in spatial data, and is able to distinguish non-linear modes. SOMs do not require prior knowledge of the data, such as distribution assumptions (e.g. normality), and so they have been applied to many areas of geoscience, such as investigating variability in the Indian Ocean^14, 45^, analysing satellite imagery^46^, pattern recognition^47^, extreme climate events^48^, geographic information systems^49^. For this study, SOMs were used to analyse spatio-seasonal malaria incidence anomaly patterns in Limpopo at the municipality level. Spatio-seasonal SOM patterns were divided into high, medium and low types.

Composite maps were made for high and low malaria incidence anomaly patterns for both the peak and pre-peak of the malaria season. By analysing incidence anomalies in the pre-peak malaria season, we were able to test whether malaria incidence occurred earlier than expected. Both peak and pre-peak seasons are considered important in terms of providing an early warning for malaria outbreaks. Since high and low composite maps showed mirroring images, we took the difference between high and low composites. This was then tested for statistical significance based on a two-tailed t test at the 10% significance level. Composite maps for precipitation, mean temperature and wind anomalies covering southern Africa were plotted at zero to three month lags. In addition, at the same lags, composites for SST anomalies in the tropical eastern Pacific west of Peru and the Indian Ocean were created to test whether there were large-scale climate phenomena, such as El Niño/La Niña and IOSD events, linked to malaria incidence patterns.

### Meteorological data

Gridded time-series climate data for precipitation, and mean temperature were obtained from Climatic Research Unit, University of East Anglia (CRU TS Version 3.23) at 0.5° × 0.5° resolution^50^. Gridded SST was obtained from NCEP/NCAR OISST^51^ and 10-m wind velocity from ECMWF reanalysis product of ERA Interim at 1° × 1° resolution^52^.

### Software

The SOM analysis was done with SOM_PAK Version 3.1^53^, and figures and all other analyses were done with R version 3.2.2^54^.

## Electronic supplementary material

Supplementary Figures
