# Supplementary material for: Seasonally lagged effects of climatic factors on malaria incidence in South Africa
Source: Sci Rep. 2017 May 29;7:2458. doi: 10.1038/s41598-017-02680-6 (PMC5447659; doi:10.1038/s41598-017-02680-6)
Supplement: Supplementary file 1 — Supplementary Figures [file 41598_2017_2680_MOESM1_ESM.doc]

**Seasonally lagged effects of climatic factors on malaria incidence in South Africa**

**Authors**

Takayoshi Ikeda*^1^**,** Swadhin K. Behera^1^, Yushi Morioka^1^, Noboru Minakawa^2^, Masahiro Hashizume^2^, Ataru Tsuzuki^2^, Rajendra Maharaj^3^, Philip Kruger^4^

**Affiliations**

[*tak.ikeda@jamstec.go.jp](mailto:*tak.ikeda@jamstec.go.jp)

1- Japan Agency for Marine-Earth Science and Technology, Yokohama Institute for Earth Sciences, 3173-25 Showa-machi, Kanazawa-ku, Yokohama, 236-0001 Japan

2- Nagasaki University, Institute of Tropical Medicine, 1-12-4 Sakamoto, Nagasaki 852-8523 Japan

3- Malaria Research Programme, Medical Research Council, Ridge Road, Durban 4067 South Africa

4- Malaria Control Programme, Limpopo Department of Health, Voortrekker Street, Tzaneen, Limpopo 0850 South Africa

Supplemental Figure Captions

Supplemental Figure S1 – Composites of a) precipitation (shade, mm month^-1^), mean temperature (contour, ºC), and wind (vector, m s^-1^) in southern Africa, and b) SST (°C) in the tropical eastern Pacific for September. Differences in composite anomalies between the high and low malaria incidence years are shown. Rows associate to lags of zero to three months. Composites at 90% confidence level are shown. Data were obtained from CRU TS 3.23. Figure was made in R version 3.2.2 (<https://cran.r-project.org/>).

Supplemental Figure S2 – Composites of precipitation (shade, mm month^-1^), mean temperature (contour, ºC), and wind (vector, m s^-1^) in southern Africa for SON at a lag of six months. Differences in composite anomalies between the high and low malaria incidence years are shown. Composites at 90% confidence level are shown. Data were obtained from CRU TS 3.23. Figure was made in R version 3.2.2 (<https://cran.r-project.org/>).

Supplemental Figure S3 – Composites of precipitation (shade, mm month^-1^), mean skin temperature (contour, ºC), and wind (vector, m s^-1^) in southern Africa for a) SON and b) DJF. Differences in composite anomalies between the high and low malaria incidence years are shown. Rows associate to lags of zero to three months. Data for precipitation and temperature were obtained from TRMM and ECMWF ERA Interim, respectively. Composites at 90% confidence level are shown. Figure was made in R version 3.2.2 (<https://cran.r-project.org/>).

Supplemental Figure S4 – Composites of precipitation (shade, mm month^-1^), mean skin temperature (contour, ºC), and wind (vector, m s-1) in southern Africa for SON at a lag of six months. Differences in composite anomalies between the high and low malaria incidence years are shown. Composites at 90% confidence level are shown. Data for precipitation and temperature were obtained from TRMM and ECMWF ERA Interim, respectively. Figure was made in R version 3.2.2 (<https://cran.r-project.org/>)..

Supplemental Figure S1

Supplemental Figure S2

Supplemental Figure S3

Supplemental Figure S4
